# Supplementary material for: Validity of Linear and Nonlinear Measures of Gait Variability to Characterize Aging Gait with a Single Lower Back Accelerometer
Source: Sensors (Basel). 2024 Nov 21;24(23):7427. doi: 10.3390/s24237427 (PMC11644259; doi:10.3390/s24237427)
Supplement: Supplementary file 1 [file sensors-24-07427-s001.zip › Supplementary_Figures.pdf]

Validity of Linear and Nonlinear Measures of Gait Variability to Characterize Aging Gait with a Single Lower Back Accelerometer

Sophia Piergiovanni and Philippe Terrier

Haute-Ecole Arc Santé, HES-SO University of Applied Sciences and Arts Western Switzerland, 2000 Neuchâtel, Switzerland

Supplementary figures

Figure S1. All participants: Histograms, scatterplots, and Pearson's correlation coefficients, all data. Page 2

Figure S2. All participants: Histograms, scatterplots, and Pearson's correlation coefficients, only normal walking data. Page 3

Figure S3. All participants: Histograms, scatterplots, and Pearson's correlation coefficients, only metronome walking data. Page 4

Figure S4. Young participants: Histograms, scatterplots, and Pearson's correlation coefficients, all data. Page 5

Figure S5. Young participants: Histograms, scatterplots, and Pearson's correlation coefficients, only normal walking data. Page 6

Figure S6. Young participants: Histograms, scatterplots, and Pearson's correlation coefficients, only metronome walking data. Page 7

Figure S7. Older participants: Histograms, scatterplots, and Pearson's correlation coefficients, all data. Page 5

Figure S8. Older participants: Histograms, scatterplots, and Pearson's correlation coefficients, only normal walking data. Page 6

Figure S9. Older participants: Histograms, scatterplots, and Pearson's correlation coefficients, only metronome walking data. Page 7

© Piergiovanni & Terrier 2024

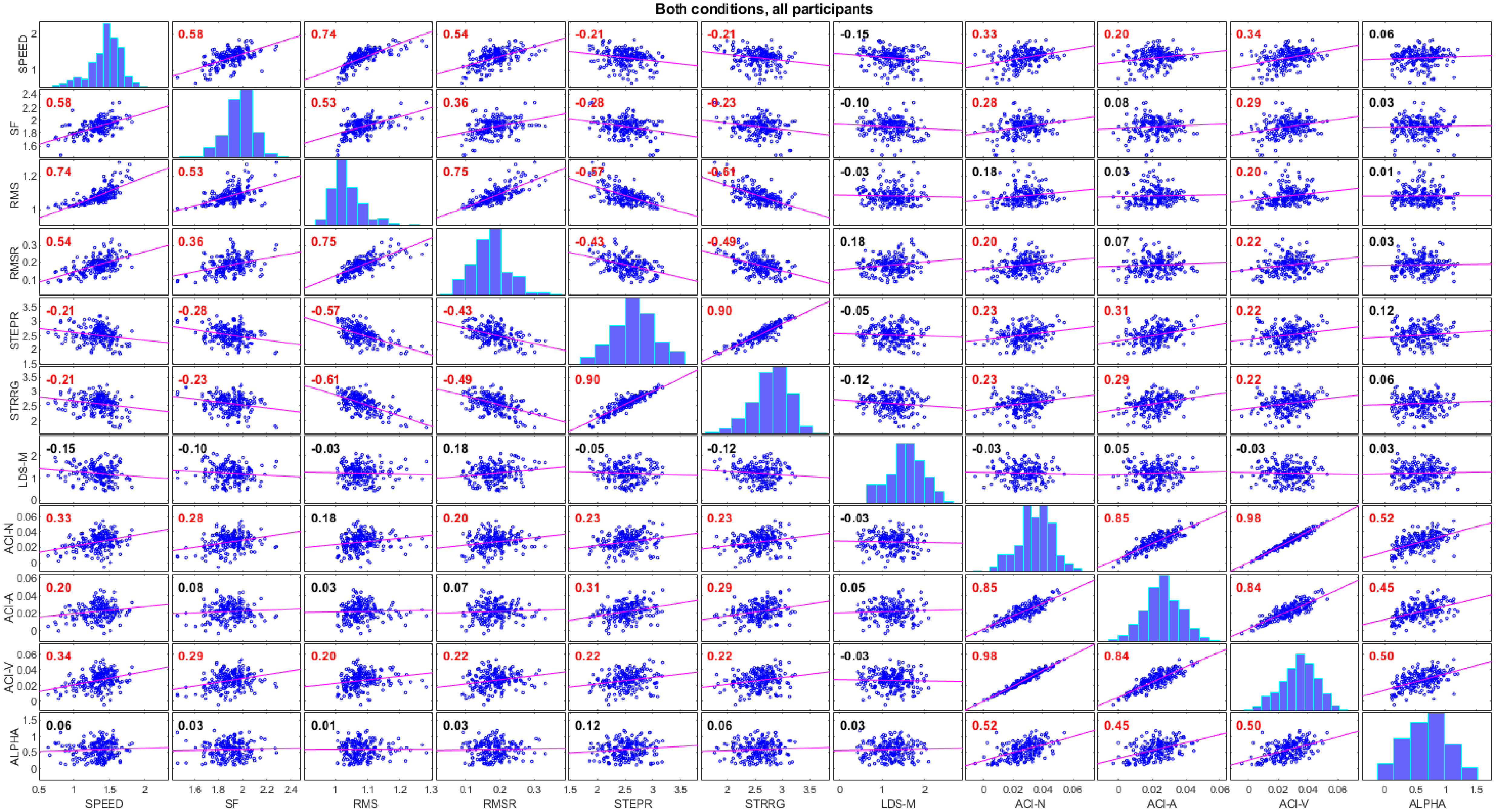

Figure S1. All participants: Histograms, scatter plots, and Pearson's correlation coefficient of gait variables for both conditions together (N=204). SF: step frequency. RMS: Root mean square (movement intensity). RMSR: RMS ratio. STEPR: Step regularity (autocorrelation function). STRRG: Stride regularity (autocorrelation function). LDS: local dynamic stability (short-term divergence). ACI: attractor complexity index (long-term divergence). Alpha: scaling exponent (detrended fluctuation analysis). N: vector norm. A: anteroposterior. V: vertical. M: mediolateral. Significant correlations (p<0.01) are highlighted in red.

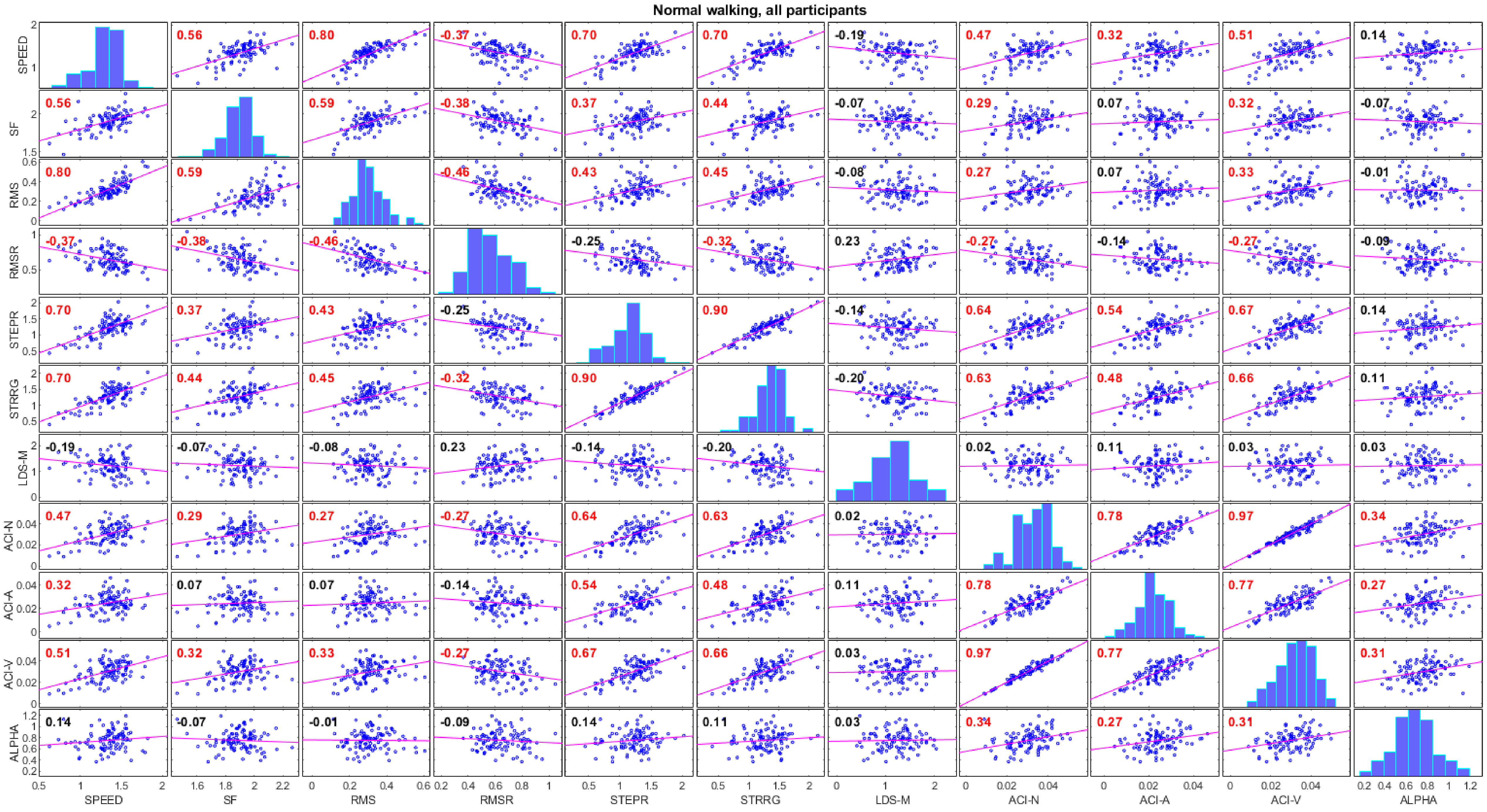

Figure S2. All participants: Histograms, scatter plots, and Pearson's correlation coefficient of gait variables for normal walking condition (N=102). SF: step frequency. RMS: Root mean square (movement intensity). RMSR: RMS ratio. STEPR: Step regularity (autocorrelation function). STRRG: Stride regularity (autocorrelation function). LDS: local dynamic stability (short-term divergence). ACI: attractor complexity index (long-term divergence). Alpha: scaling exponent (detrended fluctuation analysis). N: vector norm. A: anteroposterior. V: vertical. ML: mediolateral. Significant correlations (p<0.01) are highlighted in red.

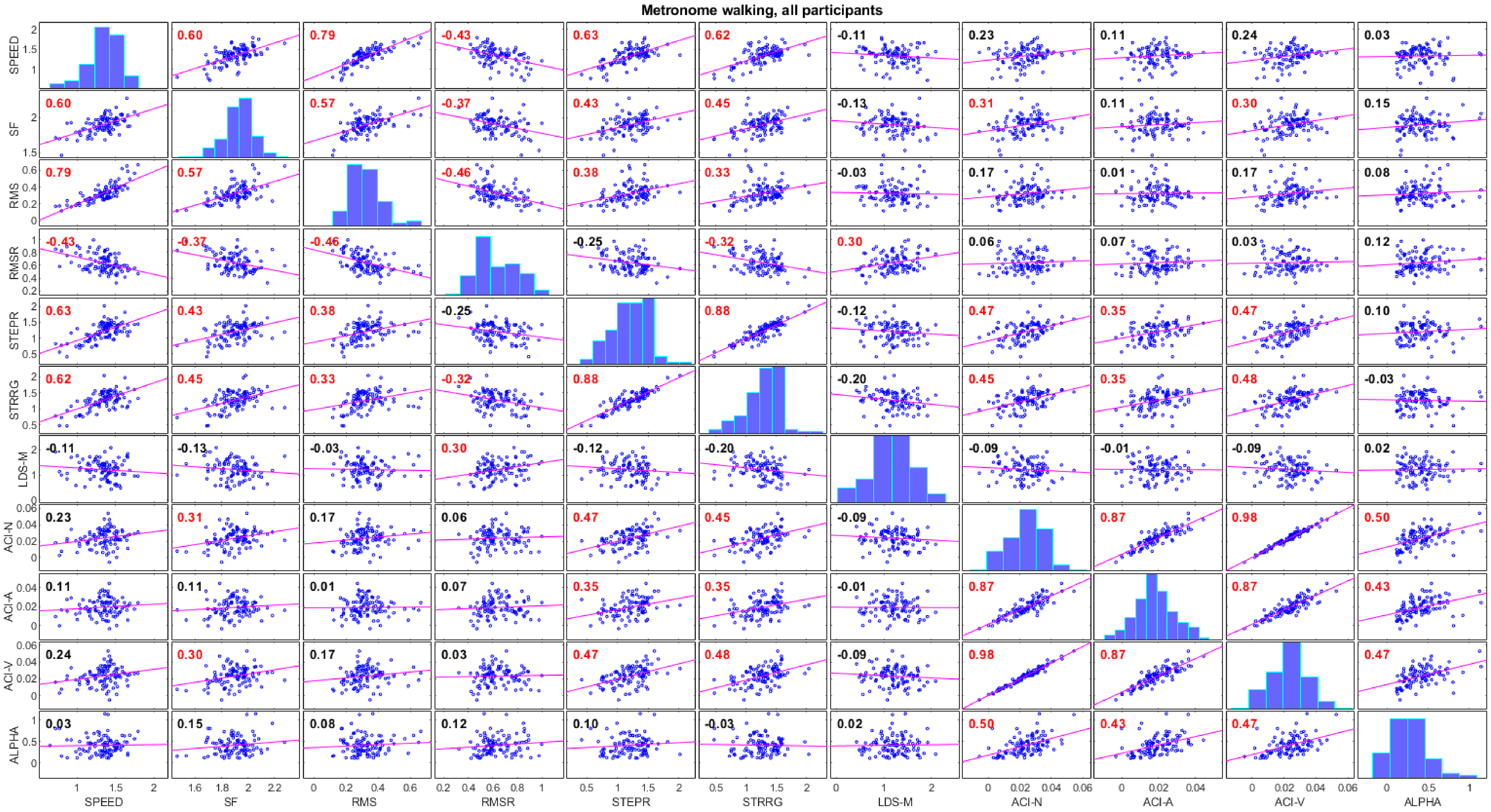

Figure S3. All participants: Histograms, scatter plots, and Pearson's correlation coefficient of gait variables for metronome walking condition (N=102). SF: step frequency. RMS: Root mean square (movement intensity). RMSR: RMS ratio. STEPR: Step regularity (autocorrelation function). STRRG: Stride regularity (autocorrelation function). LDS: local dynamic stability (short-term divergence). ACI: attractor complexity index (long-term divergence). Alpha: scaling exponent (detrended fluctuation analysis). N: vector norm. A: anteroposterior. V: vertical. M: mediolateral. Significant correlations (p<0.01) are highlighted in red.

© Piergiovanni & Terrier 2024

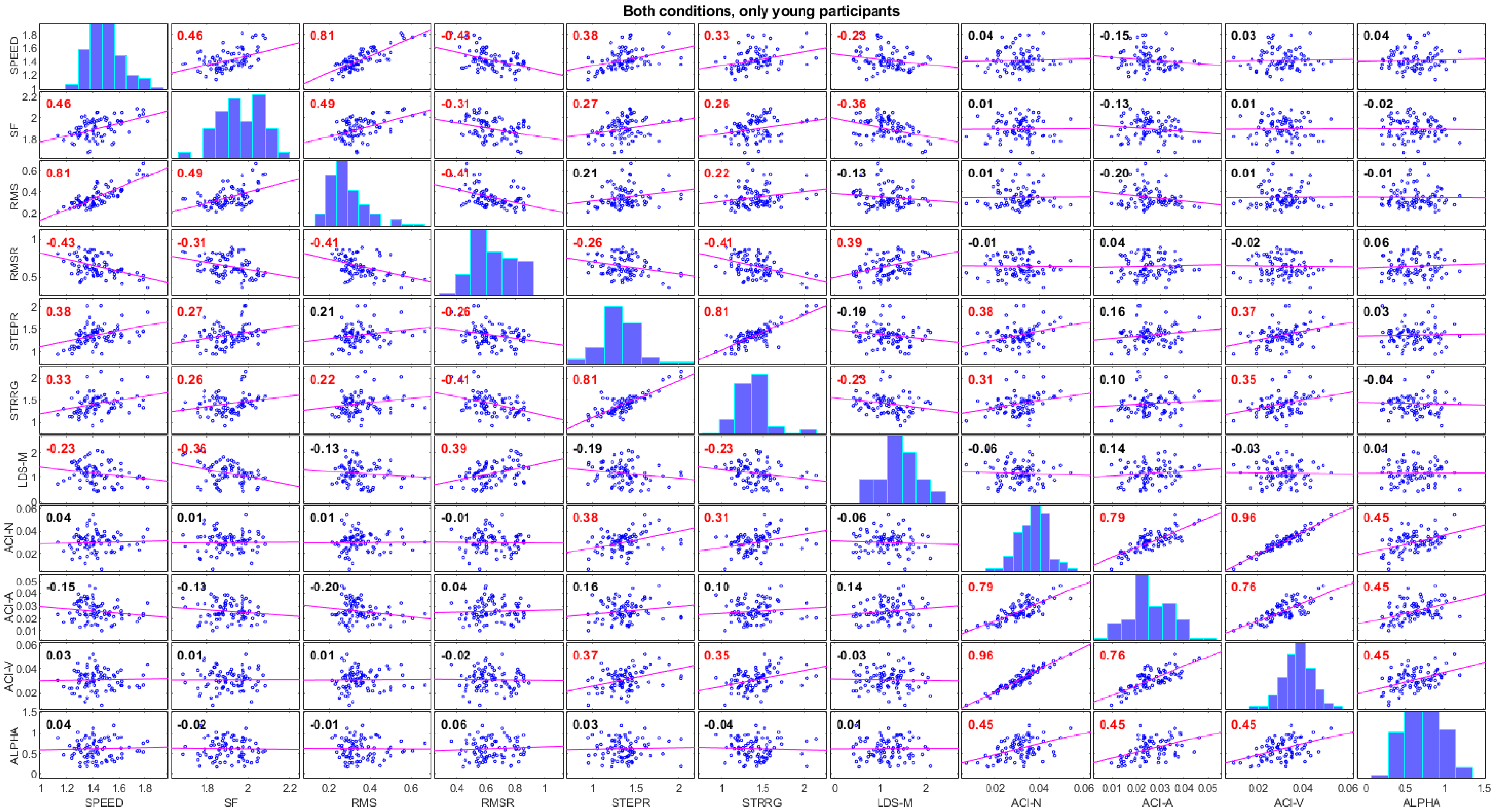

Figure S4. Young participants: Histograms, scatter plots, and Pearson's correlation coefficient of gait variables for both conditions together (N=84). SF: step frequency. RMS: Root mean square (movement intensity). RMSR: RMS ratio. STEPR: Step regularity (autocorrelation function). STRRG: Stride regularity (autocorrelation function). LDS: local dynamic stability (short-term divergence). ACI: attractor complexity index (long-term divergence). Alpha: scaling exponent (detrended fluctuation analysis). N: vector norm. A: anteroposterior. V: vertical. M: mediolateral. Significant correlations ($p<0.05$) are highlighted in red.

© Piergiovanni & Terrier 2024

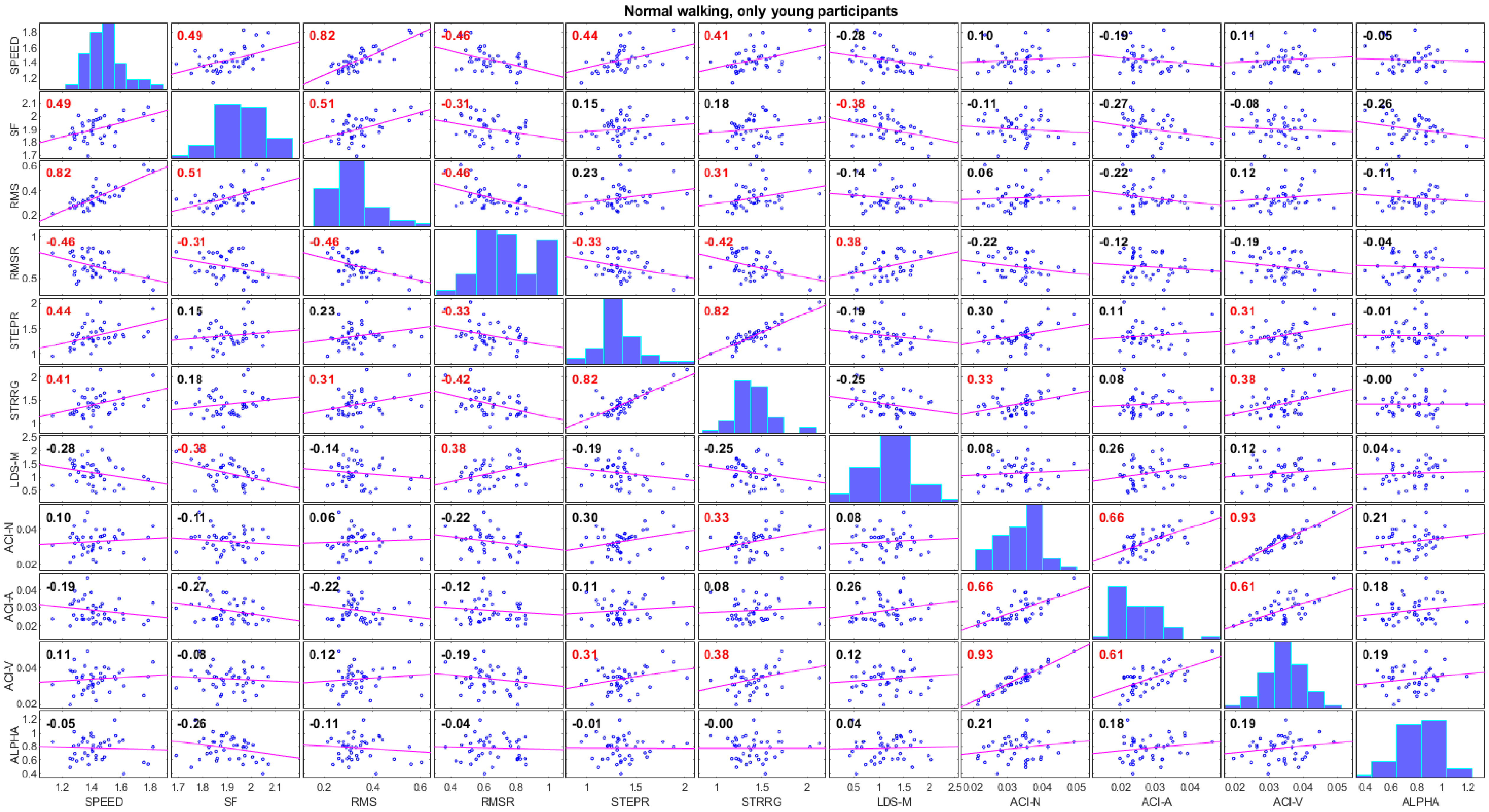

Figure S5. Young participants: Histograms, scatter plots, and Pearson's correlation coefficient of gait variables for normal walking condition (N=42). SF: step frequency. RMS: Root mean square (movement intensity). RMSR: RMS ratio. STEPR: Step regularity (autocorrelation function). STRRG: Stride regularity (autocorrelation function). LDS: local dynamic stability (short-term divergence). ACI: attractor complexity index (long-term divergence). Alpha: scaling exponent (detrended fluctuation analysis). N: vector norm. A: anteroposterior. V: vertical. M: mediolateral. Significant correlations ($p<0.05$) are highlighted in red.

CC BY

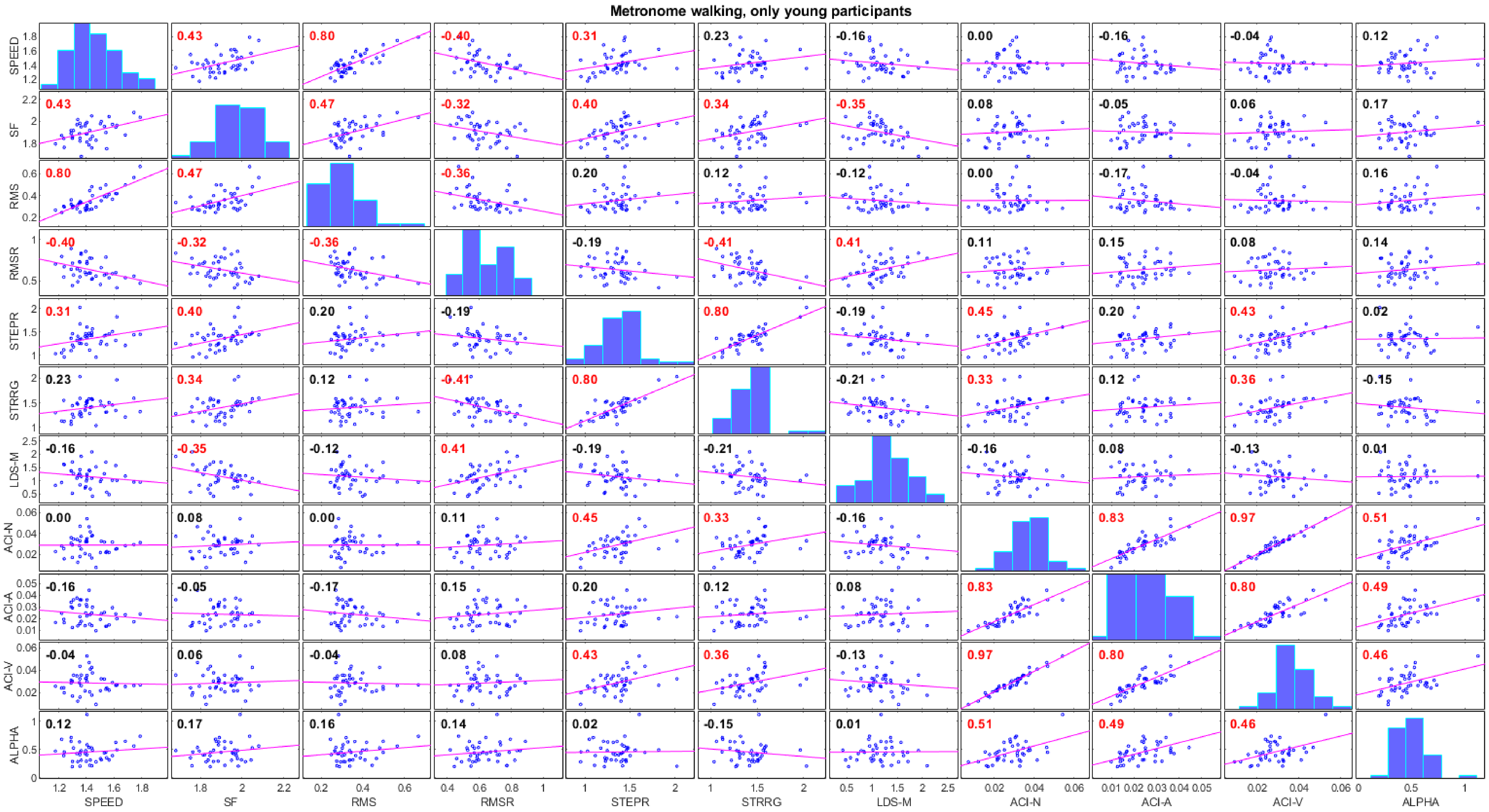

Figure S6. Young participants: Histograms, scatter plots, and Pearson's correlation coefficient of gait variables for metronome walking condition (N=42). SF: step frequency. RMS: Root mean square (movement intensity). RMSR: RMS ratio. STEPR: Step regularity (autocorrelation function). STRRG: Stride regularity (autocorrelation function). LDS: local dynamic stability (short-term divergence). ACI: attractor complexity index (long-term divergence). Alpha: scaling exponent (detrended fluctuation analysis). N: vector norm. A: anteroposterior. V: vertical. M: mediolateral. Significant correlations ($p<0.05$) are highlighted in red.

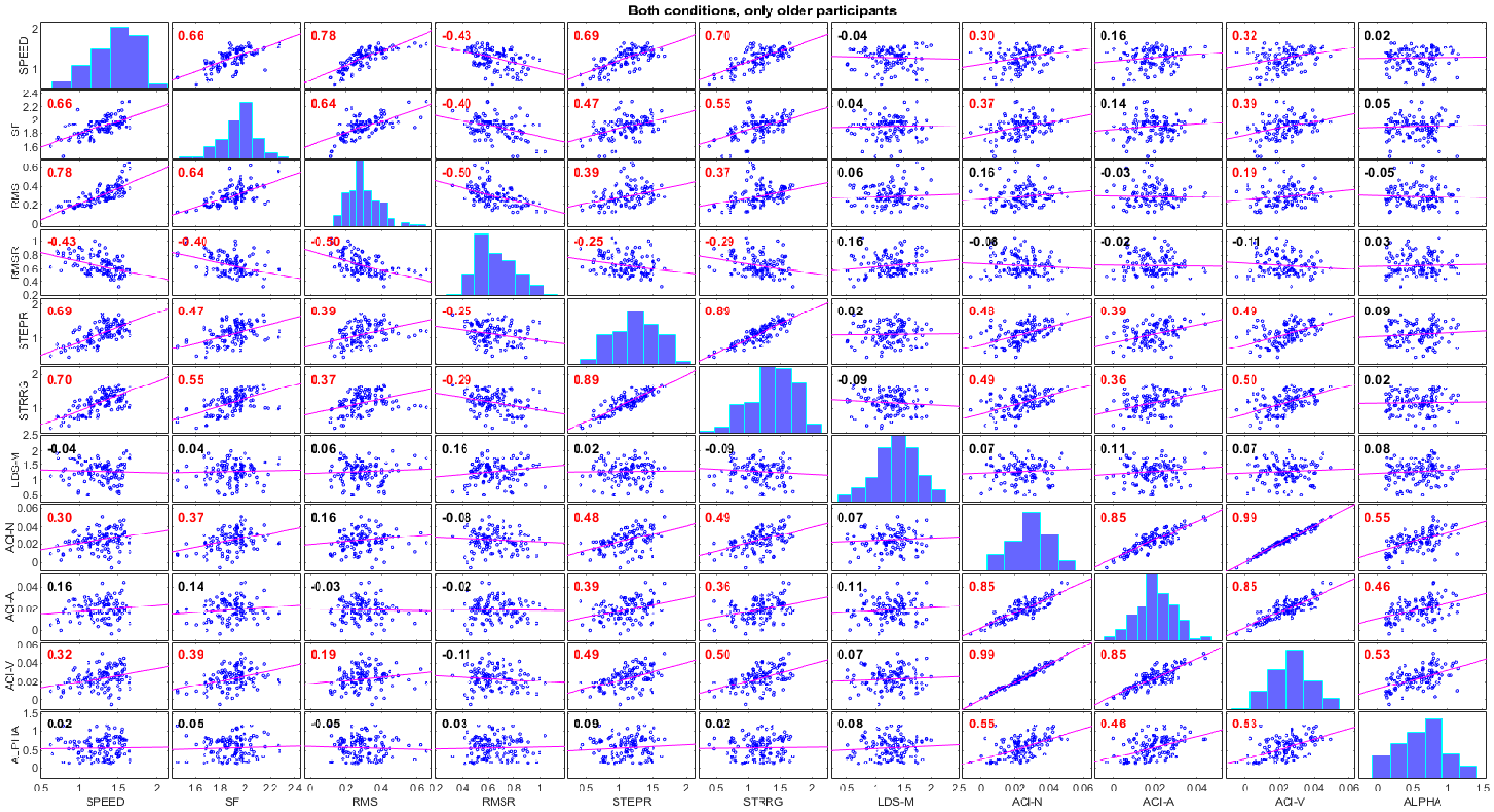

Figure S7. Older participants: Histograms, scatter plots, and Pearson's correlation coefficient of gait variables for both conditions together (N=120). SF: step frequency. RMS: Root mean square (movement intensity). RMSR: RMS ratio. STEPR: Step regularity (autocorrelation function). STRRG: Stride regularity (autocorrelation function). LDS: local dynamic stability (short-term divergence). ACI: attractor complexity index (long-term divergence). Alpha: scaling exponent (detrended fluctuation analysis). N: vector norm. A: anteroposterior. V: vertical. M: mediolateral. Significant correlations ($p<0.05$) are highlighted in red.

© Piergiovanni & Terrier 2024

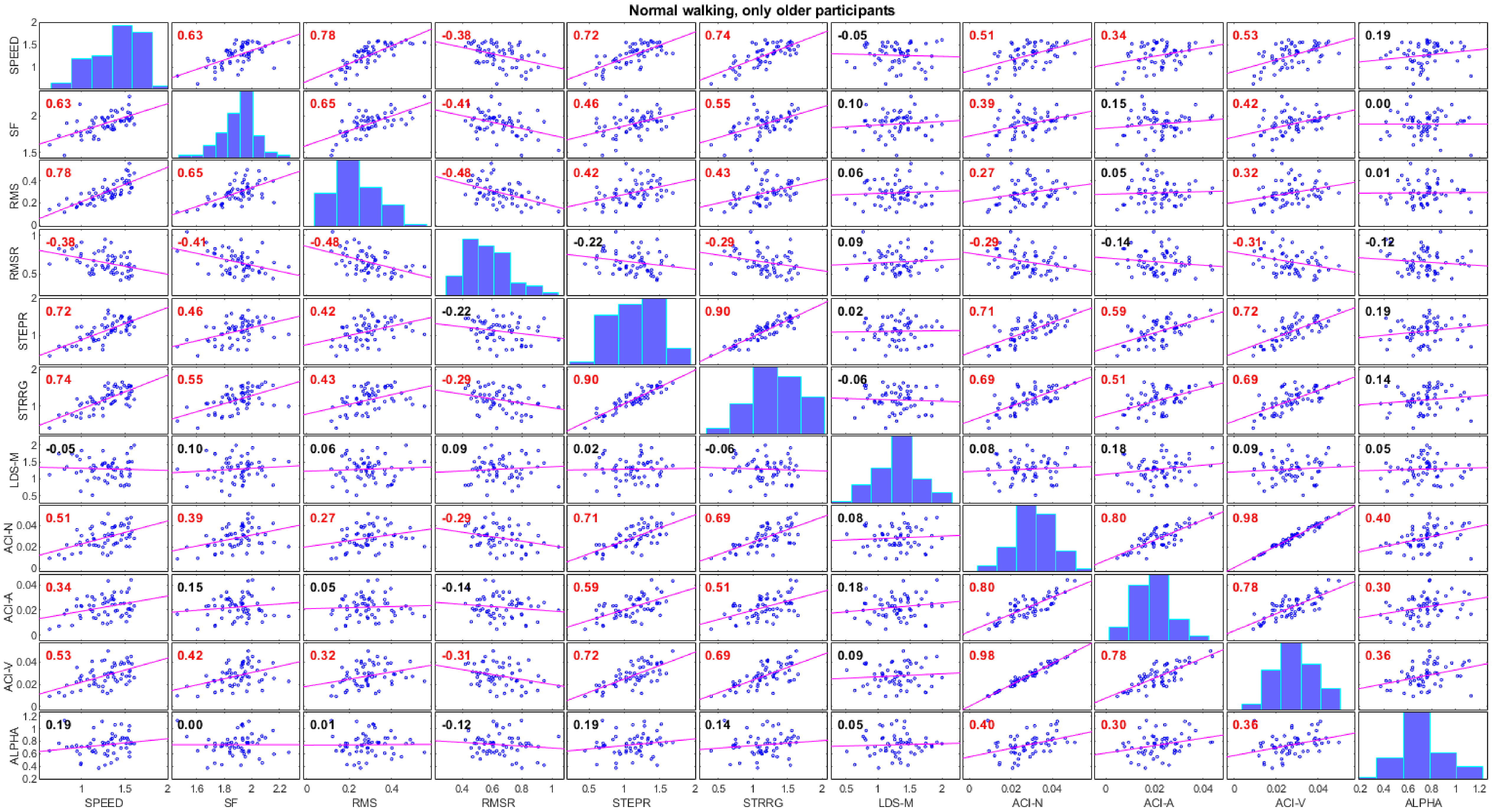

Figure S8. Older participants: Histograms, scatter plots, and Pearson's correlation coefficient of gait variables for normal walking condition (N=60). SF: step frequency. RMS: Root mean square (movement intensity). RMSR: RMS ratio. STEPR: Step regularity (autocorrelation function). STRRG: Stride regularity (autocorrelation function). LDS: local dynamic stability (short-term divergence). ACI: attractor complexity index (long-term divergence). Alpha: scaling exponent (detrended fluctuation analysis). N: vector norm. A: anteroposterior. V: vertical. M: mediolateral. Significant correlations ($p<0.05$) are highlighted in red.

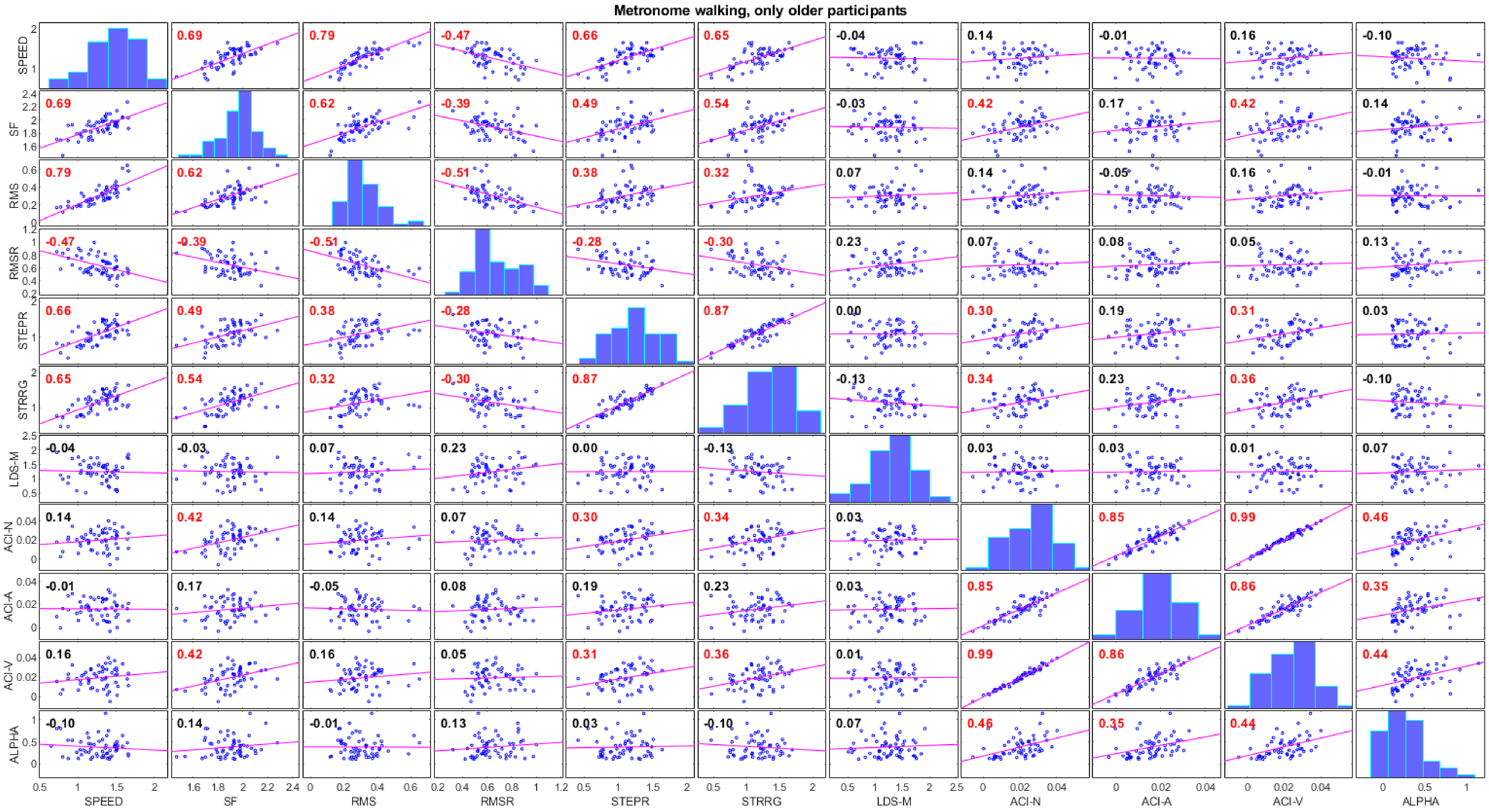

Figure S9. Older participants: Histograms, scatter plots, and Pearson's correlation coefficient of gait variables for metronome walking condition (N=60). SF: step frequency. RMS: Root mean square (movement intensity). RMSR: RMS ratio. STEPR: Step regularity (autocorrelation function). STRRG: Stride regularity (autocorrelation function). LDS: local dynamic stability (short-term divergence). ACI: attractor complexity index (long-term divergence). Alpha: scaling exponent (detrended fluctuation analysis). N: vector norm. A: anteroposterior. V: vertical. M: mediolateral. Significant correlations ($p<0.05$) are highlighted in red.

© Piergiovanni & Terrier 2024
