# Supplementary material for: Validity of Linear and Nonlinear Measures of Gait Variability to Characterize Aging Gait with a Single Lower Back Accelerometer
Source: Sensors (Basel). 2024 Nov 21;24(23):7427. doi: 10.3390/s24237427 (PMC11644259; doi:10.3390/s24237427)
Supplement: Supplementary file 1 [file sensors-24-07427-s001.zip › Supplementary_Statistics.pdf]

Validity of Linear and Nonlinear Measures of Gait Variability to Characterize Aging Gait with a Single Lower Back Accelerometer

Sophia Piergiovanni and Philippe Terrier
Haute-Ecole Arc Santé, HES-SO University of Applied Sciences and Arts Western Switzerland, 2000 Neuchâtel, Switzerland

Supplementary material

This document described detailed inferential statistics (mixed-effects linear models).

1. **Preferred walking speed** **2**
2. **Step frequency** **4**
3. **RMS (movement intensity)** **6**
4. **RMS ratio (lateral instability)** **8**
5. **Step regularity (ACF)** **10**
6. **Stride regularity (ACF)** **12**
7. **Local dynamic stability (LDS, mediolateral)** **14**
8. **Attractor complexity index (ACI, norm)** **16**
9. **Attractor complexity index (ACI, anteroposterior)** **18**
10. **Attractor complexity index (ACI, vertical)** **20**
11. **Scaling exponent (DFA)** **22**

CC BY

1. Preferred walking speed

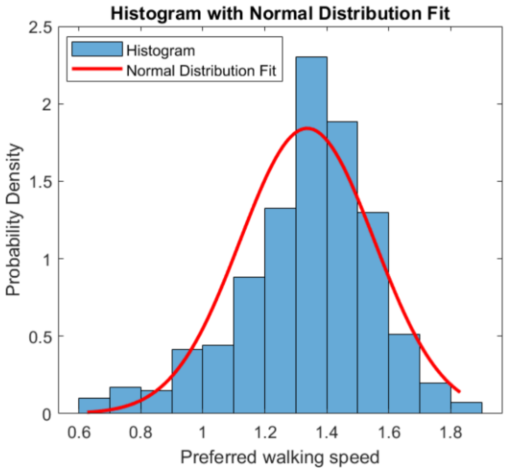

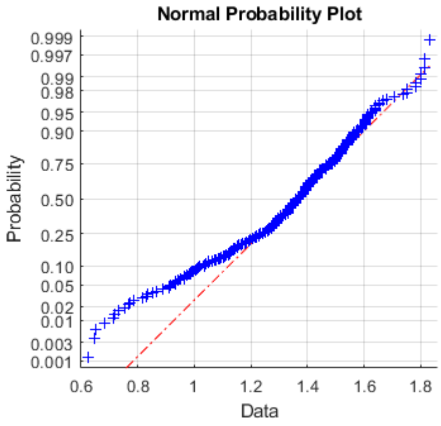

Full model

```
Linear mixed-effects model fit by ML

Model information:
    Number of observations             398
    Fixed effects coefficients           4
    Random effects coefficients        202
    Covariance parameters                4

Formula:
    speed ~ 1 + group*Conditions + (1 + Conditions | record_id)

Model fit statistics:
    AIC        BIC       LogLikelihood    Deviance
    -978.59    -946.7    497.3            -994.59
```

ANOVA marginal tests: DFMethod = 'Residual'

Term	FStat	DF1	DF2	pValue
{'(Intercept)' }	1932.2	1	394	5.3269e-154
{'group' }	17.167	1	394	4.1917e-05
{'Conditions' }	0.52748	1	394	0.4681
{'group:Conditions'}	1.0024	1	394	0.31735

Final model, no interaction

Linear mixed-effects model fit by REML

```
Model information:
    Number of observations             398
    Fixed effects coefficients           3
    Random effects coefficients        202
    Covariance parameters                4

Formula:
    speed ~ 1 + group + Conditions + (1 + Conditions | record_id)

Model fit statistics:
    AIC        BIC       LogLikelihood    Deviance
    -960.75    -932.9    487.37           -974.75
```

Fixed effects coefficients :

Name	Estimate	SE	tStat	DF	pValue
{'(Intercept)' }	1.4259	0.032372	44.047	395	**1.6937e-154**
{'group_1' }	-0.16686	0.041879	-3.9844	395	**8.056e-05**
{'Conditions_1'}	0.00029322	0.0060057	0.048824	395	0.96108

CC BY

Fixed effects, 99% CIs

Name	Lower	Upper
{'(Intercept)' }	**1.3421**	**1.5097**
{'group_1' }	**-0.2753**	**-0.0585**
{'Conditions_1'}	-0.0153	0.0158

Random effects covariance parameters :

Group: record_id (101 Levels)

Name1	Name2	Type	Estimate
{'(Intercept)' }	{'(Intercept)' }	{'std' }	0.2119
{'Conditions_1'}	{'(Intercept)' }	{'corr'}	-0.22368
{'Conditions_1'}	{'Conditions_1'}	{'std' }	0.05132

Group: Error

Name	Estimate
{'Res Std'}	0.03052

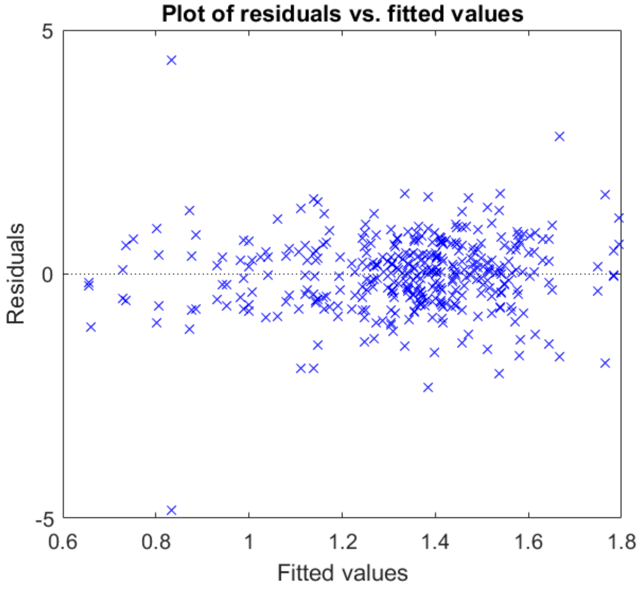

CC BY

2. Step frequency

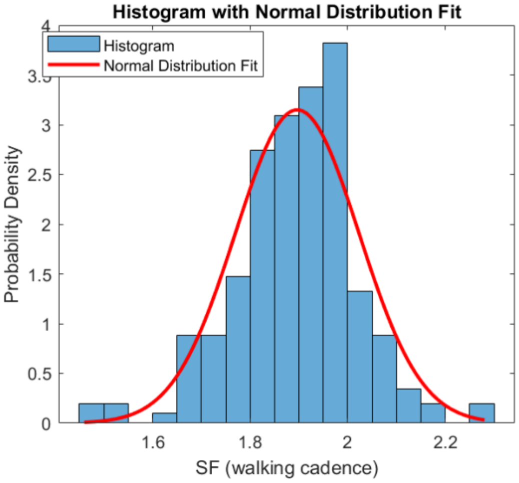

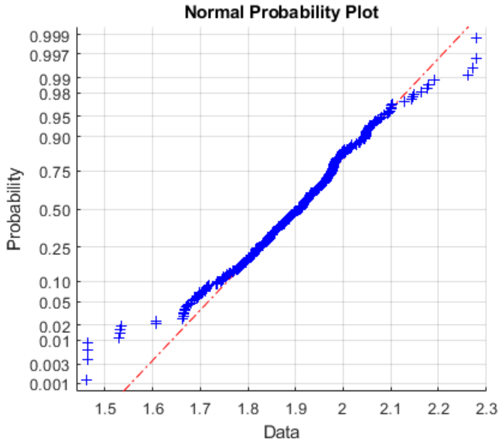

Full model

Linear mixed-effects model fit by ML

```
Model information:
    Number of observations              402
    Fixed effects coefficients            4
    Random effects coefficients         202
    Covariance parameters                 4

Formula:
    SF ~ 1 + group*Conditions + (1 + Conditions | record_id)

Model fit statistics:
    AIC         BIC         LogLikelihood    Deviance
    -1491.2     -1459.3     753.61           -1507.2
```

ANOVA marginal tests: DFMethod = 'Residual'

Term	FStat	DF1	DF2	pValue
{'(Intercept)' }	9716.5	1	398	**1.0069e-281**
{'group' }	0.3023	1	398	0.58275
{'Conditions' }	0.12272	1	398	0.72629
{'group:Conditions'}	1.1189	1	398	0.29079

Final model, no interaction

Linear mixed-effects model fit by REML

```
Model information:
    Number of observations              402
    Fixed effects coefficients            3
    Random effects coefficients         202
    Covariance parameters                 4

Formula:
    SF ~ 1 + group + Conditions + (1 + Conditions | record_id)

Model fit statistics:
    AIC         BIC         LogLikelihood    Deviance
    -1469.9     -1441.9     741.93           -1483.9
```

Fixed effects coefficients:

Name	Estimate	SE	tStat	DF	pValue
{'(Intercept)' }	1.9032	0.019489	97.657	399	**1.1532e-280**
{'group_1' }	-0.012191	0.025478	-0.47851	399	0.63255
{'Conditions_1'}	0.0020603	0.0029646	0.69496	399	0.48748

Fixed effects, 99% CIs

```
 Name                           Lower      Upper
 {'(Intercept)' }               1.8528     1.9537
 {'group_1'     }              -0.0781     0.0538
 {'Conditions_1'}              -0.0056     0.0097
```

Random effects covariance parameters:

```
Group: record_id (101 Levels)
    Name1                   Name2                   Type              Estimate
    {'(Intercept)' }        {'(Intercept)' }        {'std' }            0.12595
    {'Conditions_1'}        {'(Intercept)' }        {'corr'}          -0.035548
    {'Conditions_1'}        {'Conditions_1'}        {'std' }           0.024935

Group: Error
    Name                  Estimate
    {'Res Std'}           0.016036
```

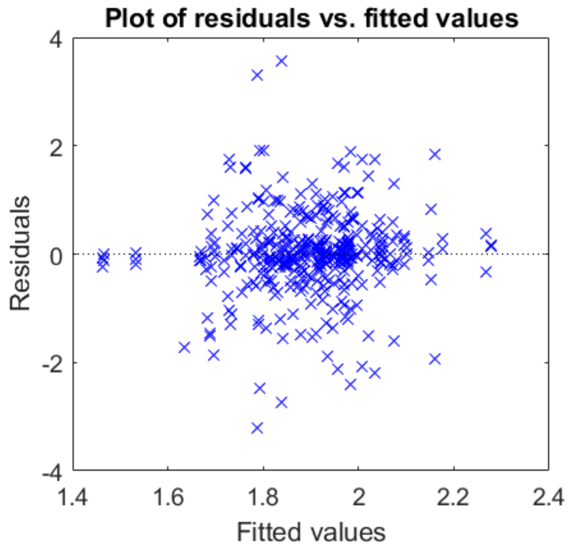

cc BY

3. RMS (movement intensity)

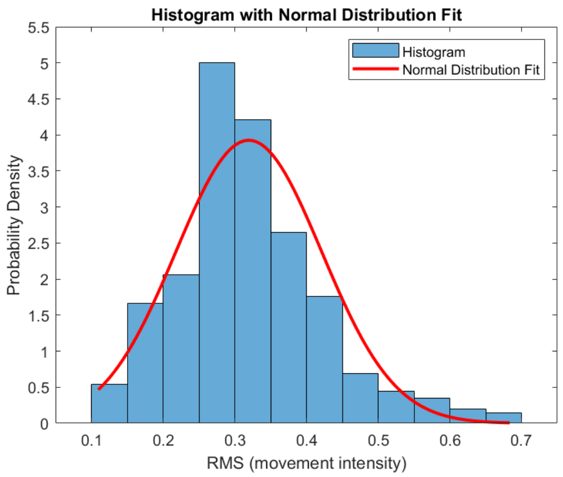

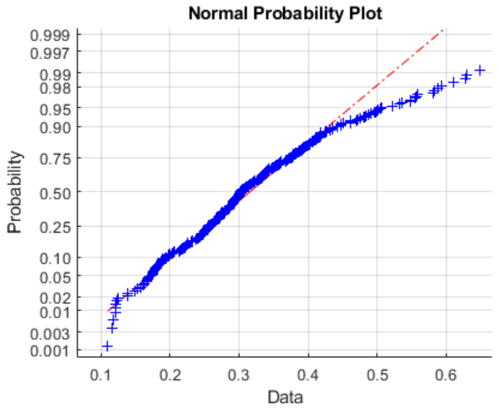

Full model

Linear mixed-effects model fit by ML

Model information:

Number of observations	402
Fixed effects coefficients	4
Random effects coefficients	202
Covariance parameters	4

Formula:
RMS_norm ~ 1 + group*Conditions + (1 + Conditions | record_id)

Model fit statistics:

AIC	BIC	LogLikelihood	Deviance
-1284.7	-1252.7	650.35	-1300.7

ANOVA marginal tests: DFMethod = 'Residual'

Term	FStat	DF1	DF2	pValue
{'(Intercept)' }	590.22	1	398	**1.2995e-80**
{'group' }	8.6846	1	398	**0.0033979**
{'Conditions' }	1.9818	1	398	0.15998
{'group:Conditions'}	1.021	1	398	0.31289

Final model, no interaction

Linear mixed-effects model fit by REML

Model information:

Number of observations	402
Fixed effects coefficients	3
Random effects coefficients	202
Covariance parameters	4

Formula:
RMS_norm ~ 1 + group + Conditions + (1 + Conditions | record_id)

Model fit statistics:

AIC	BIC	LogLikelihood	Deviance
-1413.1	-1385.1	713.53	-1427.1

Fixed effects coefficients:

Name	Estimate	SE	tStat	DF	pValue
{'(Intercept)' }	0.3459	0.014348	24.108	399	**6.9067e-80**
{'group_1' }	-0.055749	0.018763	-2.9713	399	**0.003145**
{'Conditions_1'}	0.013122	0.0039452	3.326	399	**0.00096264**

```
Fixed effects, 99% CIs
 Name                            Lower      Upper
 {'(Intercept)' }                0.3088     0.3830
 {'group_1'     }               -0.1043    -0.0072
 {'Conditions_1'}                0.0029     0.0233


Random effects covariance parameters:
Group: record_id (101 Levels)
    Name1                   Name2                   Type            Estimate
    {'(Intercept)' }        {'(Intercept)' }        {'std' }        0.092112
    {'Conditions_1'}        {'(Intercept)' }        {'corr'}         0.11179
    {'Conditions_1'}        {'Conditions_1'}        {'std' }        0.034763

Group: Error
    Name                Estimate     Lower
    {'Res Std'}         0.018655     0.016918     0.02057
```

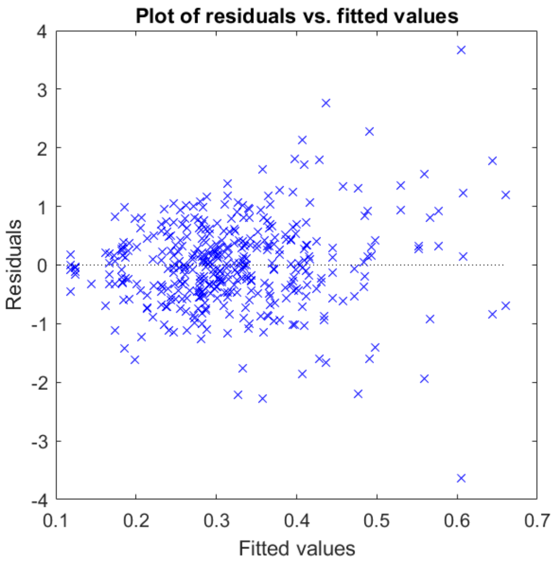

cc BY

4. RMS Ratio

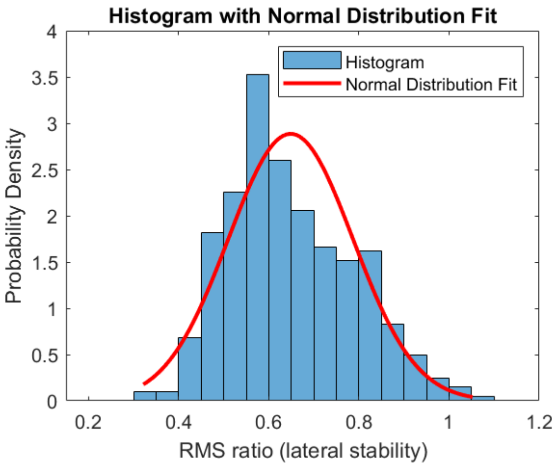

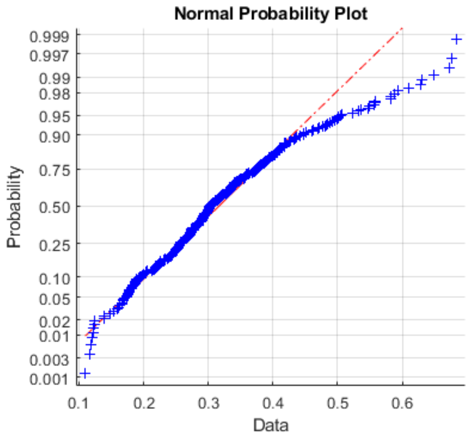

Full model

Linear mixed-effects model fit by ML

Model information:

Number of observations	402
Fixed effects coefficients	4
Random effects coefficients	202
Covariance parameters	4

Formula:

RMS_ratio_tot ~ 1 + group*Conditions + (1 + Conditions | record_id)

Model fit statistics:

AIC	BIC	LogLikelihood	Deviance
-1031.7	-999.72	523.84	-1047.7

ANOVA marginal tests: DFMethod = 'Residual'

Term	FStat	DF1	DF2	pValue
{'(Intercept)' }	981.14	1	398	1.8611e-109
{'group' }	0.16067	1	398	0.68876
{'Conditions' }	1.4279	1	398	0.23282
{'group:Conditions'}	0.34216	1	398	0.55891

Final model, no interaction

Linear mixed-effects model fit by REML

Model information:

Number of observations	402
Fixed effects coefficients	3
Random effects coefficients	202
Covariance parameters	4

Formula:

RMS_ratio_tot ~ 1 + group + Conditions + (1 + Conditions | record_id)

Model fit statistics:

AIC	BIC	LogLikelihood	Deviance
-1012.6	-984.66	513.29	-1026.6

Fixed effects coefficients:

Name	Estimate	SE	tStat	DF	pValue
{'(Intercept)' }	0.64467	0.020715	31.121	399	**8.8994e-109**
{'groupe_1' }	0.012967	0.026997	0.48031	399	0.63127
{'Conditions_1'}	-0.0063668	0.005543	-1.1486	399	0.2514

Fixed effects, 99% CIs

Name	Lower	Upper
{'(Intercept)' }	**0.4464**	**0.5030**
{'group_1' }	-0.0569	0.0828
{'Conditions_1'}	-0.0207	0.0080

Random effects covariance parameters:

Group: record_id (101 Levels)

Name1	Name2	Type	Estimate
{'(Intercept)' }	{'(Intercept)' }	{'std' }	0.13259
{'Conditions_1'}	{'(Intercept)' }	{'corr'}	-0.068118
{'Conditions_1'}	{'Conditions_1'}	{'std' }	0.042398

Group: Error

Name	Estimate
{'Res Std'}	0.035714

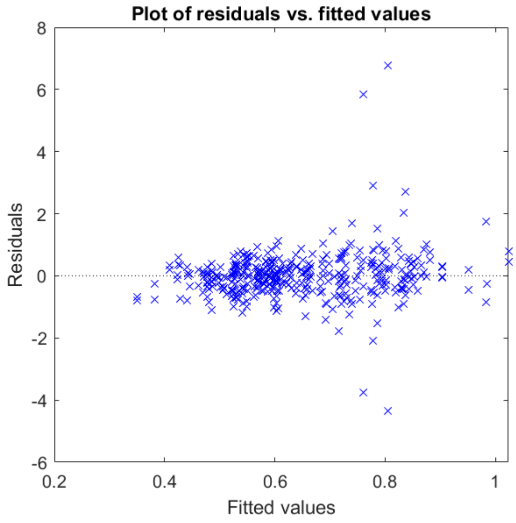

cc BY

5. Step regularity (ACF)

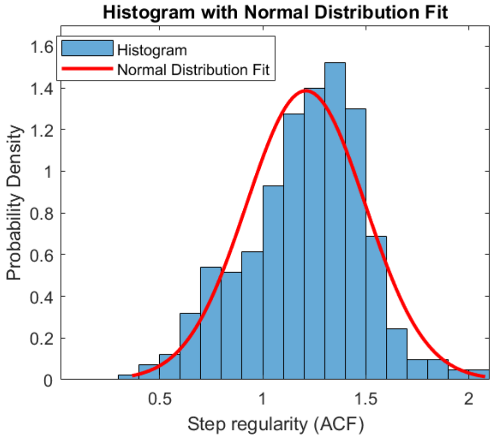

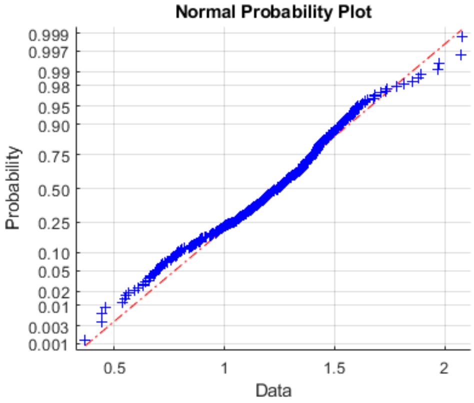

Full model

Linear mixed-effects model fit by ML

```
Model information:
    Number of observations              402
    Fixed effects coefficients            4
    Random effects coefficients         202
    Covariance parameters                 4

Formula:
    STEPVAR ~ 1 + group*Conditions + (1 + Conditions | record_id)

Model fit statistics:
    AIC        BIC        LogLikelihood    Deviance
    -495.43    -463.46    255.72           -511.43
```

ANOVA marginal tests: DFMethod = 'Residual'

Term	FStat	DF1	DF2	pValue
{'(Intercept)' }	1159.9	1	398	**5.3065e-120**
{'groupe' }	23.986	1	398	**1.414e-06**
{'Conditions' }	0.77554	1	398	0.37904
{'groupe:Conditions'}	0.065073	1	398	0.79878

Final model, no interaction

Linear mixed-effects model fit by REML

```
Model information:
    Number of observations              402
    Fixed effects coefficients            3
    Random effects coefficients         202
    Covariance parameters                 4

Formula:
    STEPVAR ~ 1 + group + Conditions + (1 + Conditions | record_id)

Model fit statistics:
    AIC        BIC        LogLikelihood    Deviance
   -480.67    -452.75     247.34           -494.67
```

Fixed effects coefficients:

Name	Estimate	SE	tStat	DF	pValue
{'(Intercept)' }	1.3642	0.039195	34.806	399	**5.8953e-123**
{'group_1' }	-0.2527	0.050017	-5.0524	399	**6.6559e-07**
{'Conditions_1'}	-0.013002	0.012342	-1.0534	399	0.29277

cc BY

```
Fixed effects, 99% CIs
Name                          Lower      Upper
 {'(Intercept)' }             1.3116     1.5189
 {'group_1'     }            -0.3794    -0.1180
 {'Conditions_1'}            -0.0432     0.0254
```

```
Random effects covariance parameters:
Group: record_id (101 Levels)
    Name1                  Name2                  Type             Estimate
    {'(Intercept)' }       {'(Intercept)' }       {'std' }          0.26151
    {'Conditions_1'}       {'(Intercept)' }       {'corr'}         -0.41812
    {'Conditions_1'}       {'Conditions_1'}       {'std' }         0.078675

Group: Error
    Name                Estimate
    {'Res Std'}          0.10681
```

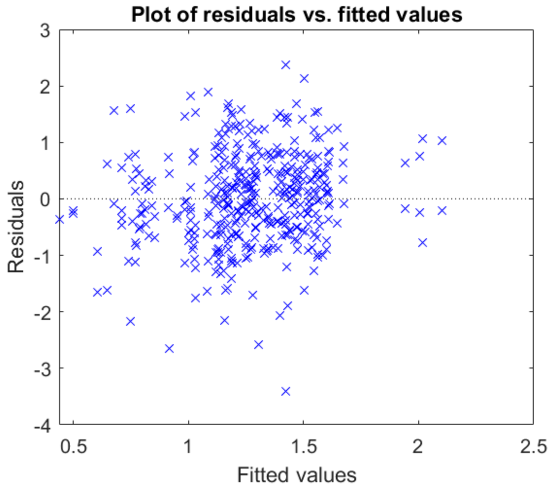

CC BY

6. Stride regularity (ACF)

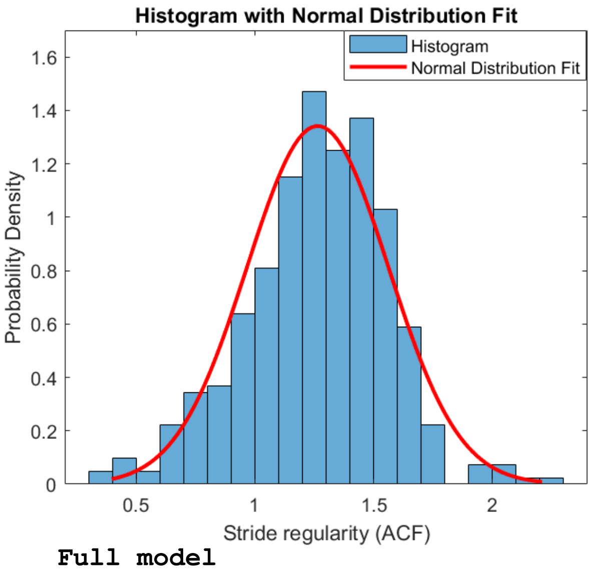

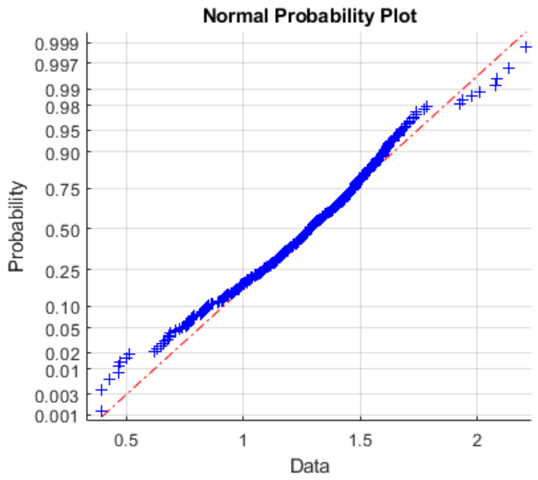

Full model

Linear mixed-effects model fit by ML

Model information:
    Number of observations 402
    Fixed effects coefficients 4
    Random effects coefficients 202
    Covariance parameters 4

Formula:
    STRIDVAR ~ 1 + group*Conditions + (1 + Conditions | record_id)

Model fit statistics:

AIC	BIC	LogLikelihood	Deviance
-289.95	-257.98	152.97	-305.95

ANOVA marginal tests: DFMethod = 'Residual'

Term	FStat	DF1	DF2	pValue
{'(Intercept)' }	1156.6	1	398	**8.0965e-120**
{'group' }	20.882	1	398	**6.5272e-06**
{'Conditions' }	0.19512	1	398	0.65893
{'groupe:Conditions'}	1.9423e-05	1	398	0.99649

Final model, no interaction

Linear mixed-effects model fit by REML

Model information:
    Number of observations 402
    Fixed effects coefficients 3
    Random effects coefficients 202
    Covariance parameters 4

Formula:
    STRIDVAR ~ 1 + group + Conditions + (1 + Conditions | record_id)

Model fit statistics:

AIC	BIC	LogLikelihood	Deviance
-470.59	-442.67	242.3	-484.59

Fixed effects coefficients:

Name	Estimate	SE	tStat	DF	pValue
{'(Intercept)' }	1.4153	0.040041	35.345	399	**5.7109e-125**
{'group_1' }	-0.24871	0.050484	-4.9265	399	**1.2287e-06**
{'Conditions_1'}	-0.0089223	0.013256	-0.6731	399	0.50128

Fixed effects, 99% CIs

```
Name                          Lower     Upper
 {'(Intercept)' }             1.7422    1.8891
 {'group_1'    }             -0.2948   -0.1094
 {'Conditions_1'}            -0.0514    0.0101
```

Random effects covariance parameters:

```
Group: record_id (101 Levels)
    Name1                  Name2                  Type           Estimate
    {'(Intercept)' }       {'(Intercept)' }       {'std' }        0.18385
    {'Conditions_1'}       {'(Intercept)' }       {'corr'}       -0.35534
    {'Conditions_1'}       {'Conditions_1'}       {'std' }       0.085996

Group: Error
    Name                 Estimate
    {'Res Std'}          0.082135
```

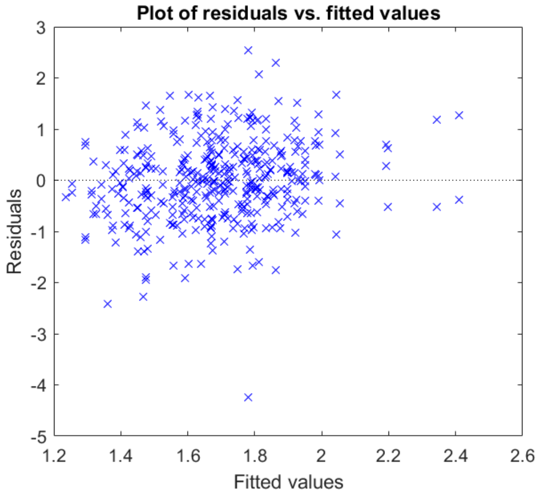

cc BY

7. Local dynamic stability (LDS, mediolateral)

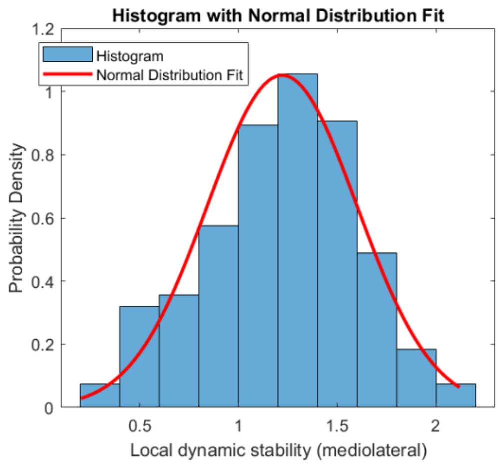

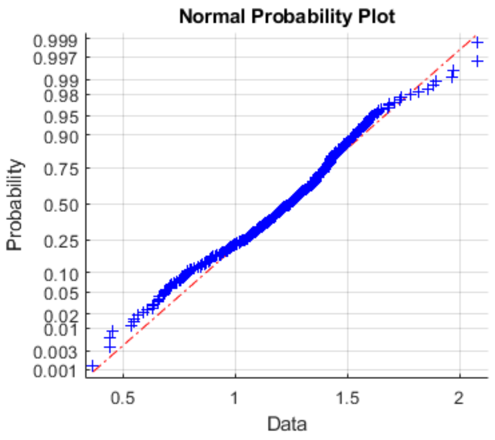

Full model

Linear mixed-effects model fit by ML

Model information:

Number of observations	402
Fixed effects coefficients	4
Random effects coefficients	202
Covariance parameters	4

Formula:
```
LDS_z ~ 1 + group*Conditions + (1 + Conditions | record_id)
```

Model fit statistics:

AIC	BIC	LogLikelihood	Deviance
27.375	59.346	-5.6874	11.375

ANOVA marginal tests: DFMethod = 'Residual'

Term	FStat	DF1	DF2	pValue
{'(Intercept)' }	419.57	1	398	**3.3841e-64**
{'group' }	3.7066	1	398	0.054911
{'Conditions' }	0.16786	1	398	0.68224
{'group:Conditions'}	1.3315	1	398	0.24924

Final model, no interaction

Linear mixed-effects model fit by REML

Model information:

Number of observations	402
Fixed effects coefficients	3
Random effects coefficients	202
Covariance parameters	4

Formula:
```
LDS_z ~ 1 + group + Conditions + (1 + Conditions | record_id)
```

Model fit statistics:

AIC	BIC	LogLikelihood	Deviance
40.846	68.768	-13.423	26.846

Fixed effects coefficients:

Name	Estimate	SE	tStat	DF	pValue
{'(Intercept)' }	1.1641	0.054414	21.393	399	3.4167e-68
{'group_1' }	0.11096	0.069138	1.6049	399	0.10931
{'Conditions_1'}	-0.016549	0.023102	-0.71635	399	0.47419

```
Fixed effects, 99% CIs
Name                            Lower      Upper
 {'(Intercept)' }               1.0233     1.3049
 {'group_1'     }              -0.0680     0.2899
 {'Conditions_1'}              -0.0763     0.0432


Random effects covariance parameters:
Group: record_id (101 Levels)
    Name1                  Name2                  Type             Estimate
    {'(Intercept)' }       {'(Intercept)' }       {'std' }          0.35017
    {'Conditions_1'}       {'(Intercept)' }       {'corr'}         -0.30586
    {'Conditions_1'}       {'Conditions_1'}       {'std' }          0.17342

Group: Error
    Name                 Estimate
    {'Res Std'}          0.15285
```

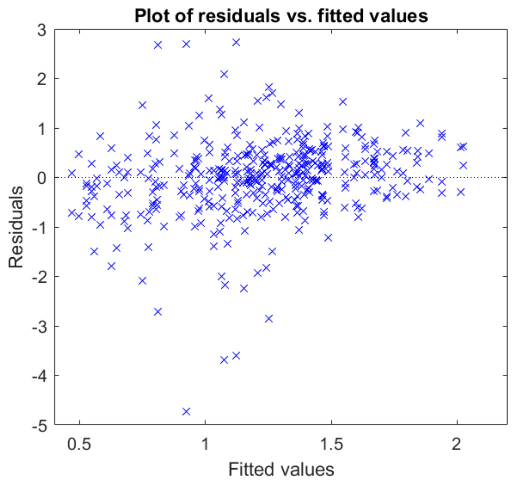

cc BY

8. Attractor complexity index (ACI, norm)

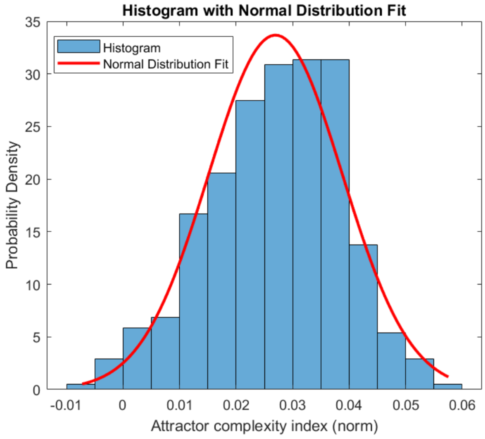

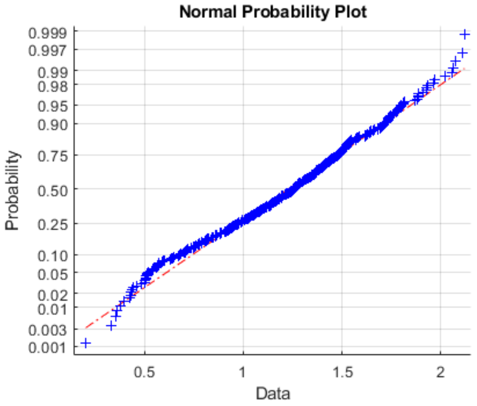

Full model

Linear mixed-effects model fit by ML

Model information:

Number of observations	402
Fixed effects coefficients	4
Random effects coefficients	202
Covariance parameters	4

Formula:
ACI_N ~ 1 + group*Conditions + (1 + Conditions | record_id)

Model fit statistics:

AIC	BIC	LogLikelihood	Deviance
-2540.4	-2508.5	1278.2	-2556.4

ANOVA marginal tests: DFMethod = 'Residual'

Term	FStat	DF1	DF2	pValue
{'(Intercept)' }	632.76	1	398	**2.9177e-84**
{'group' }	7.5489	1	398	**0.0062776**
{'Conditions' }	6.769	1	398	**0.0096215**
{'group:Conditions'}	3.4709	1	398	0.063195

Final model, no interaction

Linear mixed-effects model fit by REML

Model information:

Number of observations	402
Fixed effects coefficients	3
Random effects coefficients	202
Covariance parameters	4

Formula:
ACI_N ~ 1 + group + Conditions + (1 + Conditions | record_id)

Model fit statistics:

AIC	BIC	LogLikelihood	Deviance
-2503.5	-2475.5	1258.7	-2517.5

Fixed effects coefficients:

Name	Estimate	SE	tStat	DF	pValue
{'(Intercept)' }	0.033973	0.0012278	27.669	399	**7.6481e-95**
{'group_1' }	-0.0063218	0.0015035	-4.2048	399	**3.2284e-05**
{'Conditions_1'}	-0.0066677	0.0010966	-6.0802	399	**2.8097e-09**

```
Fixed effects, 99% CIs
Name                          Lower     Upper
{'(Intercept)' }              0.0308    0.0372
{'group_1'     }             -0.0102   -0.0024
{'Conditions_1'              -0.0095   -0.0038
```

```
Random effects covariance parameters (95% CIs):
Group: record_id (101 Levels)
    Name1                   Name2                   Type            Estimate
    {'(Intercept)' }        {'(Intercept)' }        {'std' }        0.0064732
    {'Conditions_1'}        {'(Intercept)' }        {'corr'}         -0.31906
    {'Conditions_1'}        {'Conditions_1'}        {'std' }        0.0074596

Group: Error
    Name                Estimate
    {'Res Std'}         0.008057
```

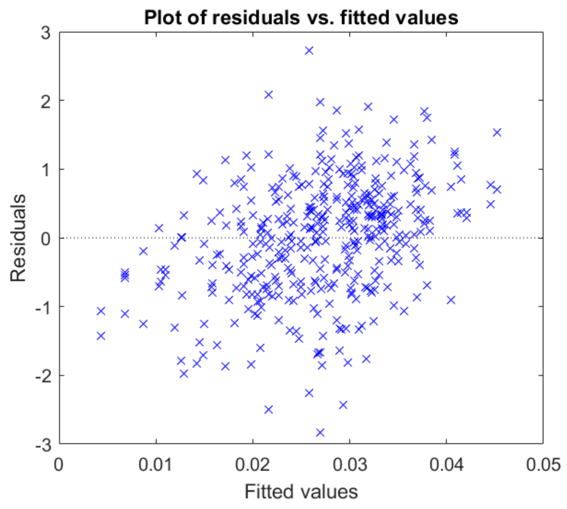

CC BY

9. Attractor complexity index (ACI, anteroposterior)

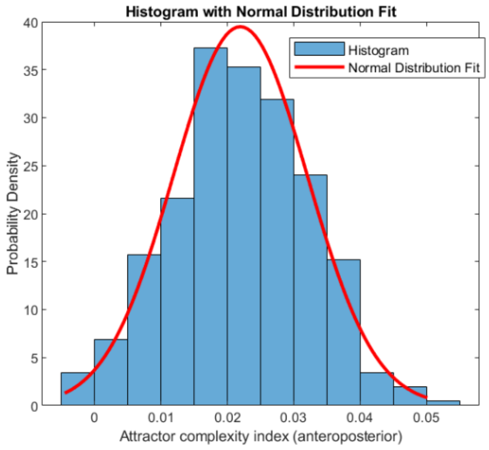

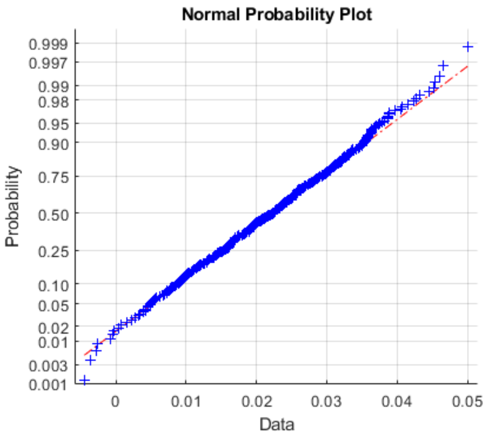

Full model

Linear mixed-effects model fit by ML

```
Model information:
    Number of observations             402
    Fixed effects coefficients           4
    Random effects coefficients        202
    Covariance parameters                4

Formula:
    ACI_x ~ 1 + group*Conditions + (1 + Conditions | record_id)

Model fit statistics:
    AIC        BIC        LogLikelihood    Deviance
    -2703.7    -2671.8    1359.9           -2719.7
```

ANOVA marginal tests: DFMethod = 'Residual'

Term	FStat	DF1	DF2	pValue
{'(Intercept)' }	581.04	1	398	**8.3484e-80**
{'group' }	15.239	1	398	**0.00011125**
{'Conditions' }	8.7554	1	398	**0.0032713**
{'group:Conditions'}	1.2095	1	398	0.2721

Final model, no interaction

Linear mixed-effects model fit by REML

```
Model information:
    Number of observations             402
    Fixed effects coefficients           3
    Random effects coefficients        202
    Covariance parameters                4

Formula:
    ACI_x ~ 1 + group + Conditions + (1 + Conditions | record_id)

Model fit statistics:
    AIC        BIC        LogLikelihood    Deviance
    -2668.1    -2640.2    1341.1           -2682.1
```

Fixed effects coefficients:

Name	Estimate	SE	tStat	DF	pValue
{'(Intercept)' }	0.028658	0.0010785	26.573	399	**2.699e-90**
{'group_1' }	-0.0068512	0.0013089	-5.2342	399	**2.6852e-07**
{'Conditions_1'}	-0.0054531	0.00093951	-5.8042	399	**1.3199e-08**

CC BY

Fixed effects, 99% CIs

```
Name                          Lower     Upper
 {'(Intercept)' }             0.0259    0.0314
 {'group_1'     }            -0.0102   -0.0035
 {'Conditions_1'}            -0.0079   -0.0030
```

Random effects covariance parameters:

```
Group: record_id (101 Levels)
    Name1                   Name2                   Type            Estimate
    {'(Intercept)' }        {'(Intercept)' }        {'std' }        0.0062709
    {'Conditions_1'}        {'(Intercept)' }        {'corr'}         -0.42899
    {'Conditions_1'}        {'Conditions_1'}        {'std' }        0.0070939

Group: Error
    Name                Estimate
    {'Res Std'}         0.0061798
```

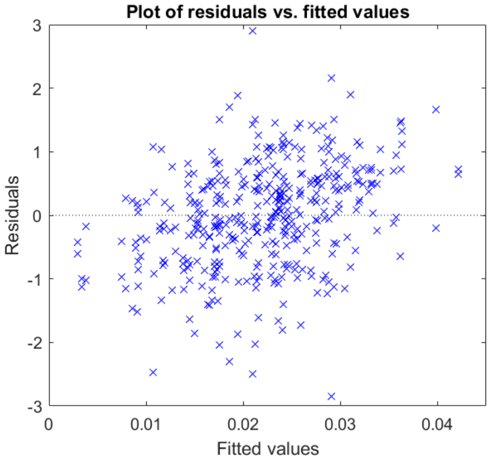

BY

10. Attractor complexity index (ACI, vertical)

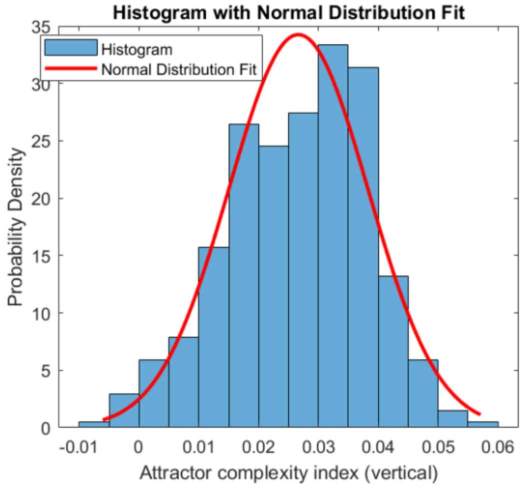

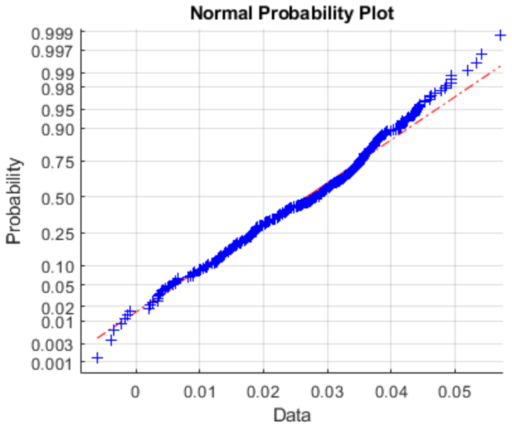

Full model

Linear mixed-effects model fit by ML

Model information:

Number of observations	402
Fixed effects coefficients	4
Random effects coefficients	202
Covariance parameters	4

Formula:

ACI_y ~ 1 + group*Conditions + (1 + Conditions | record_id)

Model fit statistics:

AIC	BIC	LogLikelihood	Deviance
-2570.3	-2538.3	1293.2	-2586.3

ANOVA marginal tests: DFMethod = 'Residual'

Term	FStat	DF1	DF2	pValue
{'(Intercept)' }	663.24	1	398	**8.7675e-87**
{'group' }	12.187	1	398	**0.00053503**
{'Conditions' }	9.4427	1	398	**0.0022658**
{'group:Conditions'}	1.9735	1	398	0.16085

Final model, no interaction

Linear mixed-effects model fit by REML

Model information:

Number of observations	402
Fixed effects coefficients	3
Random effects coefficients	202
Covariance parameters	4

Formula:

ACI_y ~ 1 + group + Conditions + (1 + Conditions | record_id)

Model fit statistics:

AIC	BIC	LogLikelihood	Deviance
-2534.7	-2506.8	1274.3	-2548.7

Fixed effects coefficients:

Name	Estimate	SE	tStat	DF	pValue
{'(Intercept)' }	0.034119	0.0012119	28.154	399	**7.7802e-97**
{'group_1' }	-0.0071215	0.0014849	-4.7958	399	**2.2921e-06**
{'Conditions_1'}	-0.0066691	0.0010569	-6.31	399	**7.4253e-10**

```
Fixed effects, 99% CIs
Name                          Lower     Upper
 {'(Intercept)' }             0.0310    0.0373
 {'group_1'     }            -0.0110   -0.0033
 {'Conditions_1'}            -0.0094   -0.0039


Random effects covariance parameters:
Group: record_id (101 Levels)
    Name1                  Name2                  Type           Estimate
    {'(Intercept)' }       {'(Intercept)' }       {'std' }       0.0065699
    {'Conditions_1'}       {'(Intercept)' }       {'corr'}      -0.33537
    {'Conditions_1'}       {'Conditions_1'}       {'std' }       0.0073242

Group: Erro
    Name              Estimate
    {'Res Std'}       0.0076381
```

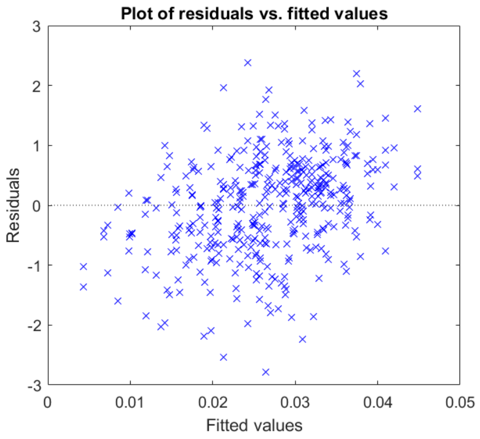

cc BY

11. Scaling exponent (DFA)

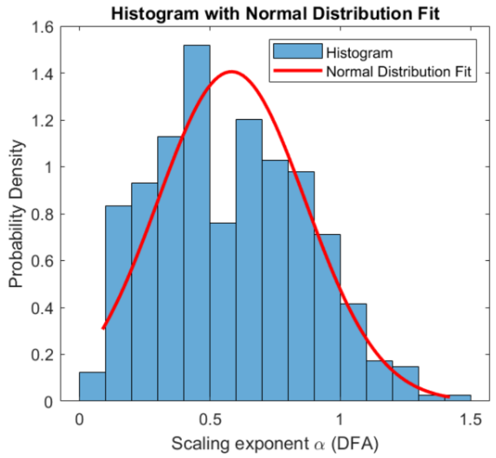

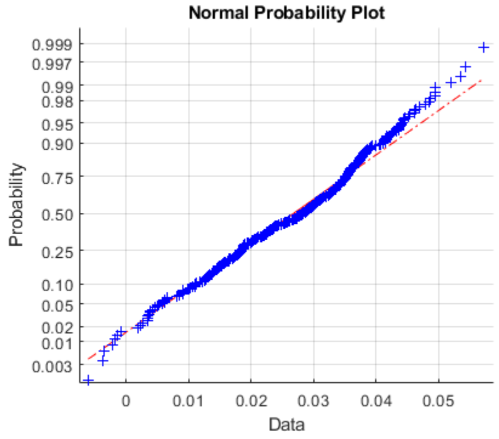

Full model

```
Linear mixed-effects model fit by ML

Model information:
    Number of observations             408
    Fixed effects coefficients           4
    Random effects coefficients        204
    Covariance parameters                4

Formula:
    alpha ~ 1 + group*Conditions + (1 + Conditions | record_id)

Model fit statistics:
    AIC        BIC        LogLikelihood    Deviance
    -67.052    -34.962    41.526           -83.052

ANOVA marginal tests: DFMethod = 'Residual'

    Term                          FStat      DF1    DF2    pValue
    {'(Intercept)'      }         872.67     1      404    5.5198e-103
    {'group'            }        0.88776     1      404        0.34665
    {'Conditions'       }         54.129     1      404     1.0572e-12
    {'group:Conditions'}         0.44581     1      404        0.50471
```

Final model, no interaction

```
Linear mixed-effects model fit by REML

Model information:
    Number of observations             408
    Fixed effects coefficients           3
    Random effects coefficients        204
    Covariance parameters                4

Formula:
    alpha ~ 1 + group + Conditions + (1 + Conditions | record_id)

Model fit statistics:
    AIC       BIC        LogLikelihood    Deviance
    -50.76    -22.732    32.38            -64.76

Fixed effects coefficients:
    Name                   Estimate     SE          tStat      DF     pValue
    {'(Intercept)' }        0.77801     0.022419     34.704    405    2.1066e-123
    {'group_1'     }      -0.047379     0.025144    -1.8843    405    0.060236
    {'Conditions_1'}       -0.33499     0.027511    -12.177    405    2.7921e-29
```

```
Fixed effects, 99% CIs
Name                         Lower      Upper
 {'(Intercept)' }             0.7200     0.8360
 {'group_1'     }            -0.1125     0.0177
 {'Conditions_1'}            -0.4062    -0.2638
```

```
Random effects covariance parameters (95% CIs):
Group: record_id (102 Levels)
    Name1                   Name2                   Type            Estimate
    {'(Intercept)' }        {'(Intercept)' }        {'std' }         0.11065
    {'Conditions_1'}        {'(Intercept)' }        {'corr'}        -0.66403
    {'Conditions_1'}        {'Conditions_1'}        {'std' }         0.20924

Group: Error
    Name                Estimate
    {'Res Std'}         0.1828
```

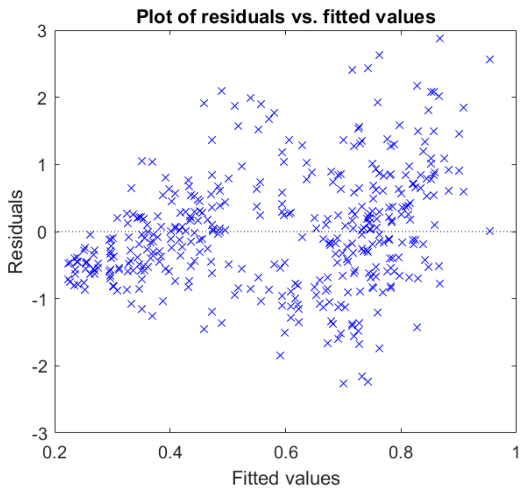

CC BY
